# Episiotomy: Are Indian Obstetricians Getting the Angle Right?

**DOI:** 10.1007/s13224-023-01871-8

**Published:** 2023-11-28

**Authors:** Nicola Adanna Okeahialam, Abdul H. Sultan

**Affiliations:** 1 Croydon University Hospital, 530 London Rd, Thornton Heath, CR7 7YE UK; 2 St George’s University of London, London, UK

**Keywords:** Mediolateral episiotomy, Perineal trauma, Obstetric anal sphincter injury, Anal incontinence, Medical education

## Abstract

**Background:**

A mediolateral episiotomy is recommended when indicated at a 60° angle at crowning, to avoid obstetric anal sphincter injuries (OASIs) by episiotomies angled too close or distant to the anus. This study surveyed obstetricians in India regarding the recommended episiotomy angle and their ability to correctly draw the angle.

**Methods:**

Workshops were conducted in India to share knowledge in the prevention and repair of OASIs. A questionnaire was distributed prior to the workshop. Participants were asked to describe the recommended episiotomy angle and to draw this on a paper replica of the perineum. The intra-class correlation coefficient (ICC) was calculated to assess the inter-rater reliability between the angle stated and drawn. A 2° difference was deemed acceptable. Standard errors of measurement (SEM) were calculated to measure the range of error of each measurement.

**Results:**

One hundred and forty doctors participated. 47.9% described the angle of an episiotomy to be 60°. Only 2.2% drew an angle of 60°, but 8.7% (*n* = 12) drew between 58 and 62°. Only 5.8% (*n* = 6) of doctors correctly drew the episiotomy angle they described. There was poor agreement ICC = 0.18 (− 0.01 to 0.36) with a SEM of ± 12.2°

**Conclusions:**

Knowledge surrounding the recommended episiotomy angle is lacking. Doctors are failing to estimate their desired episiotomy angle. This highlights the need for national guidelines, the creation and validation of structured training programmes to improve accuracy, or using fixed-angle devices such as the EPISCISSORS-60 or other proven measurement aids to minimise preventable harm due to human error.

## Introduction

**Fig. 1:**
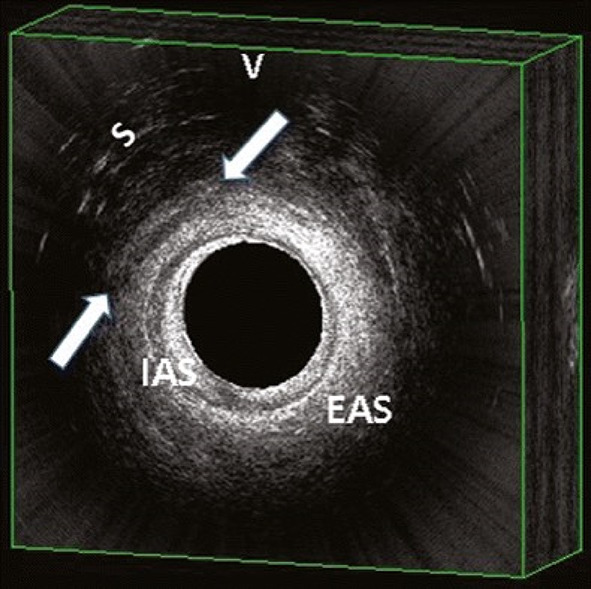
An endoanal ultrasound picture showing an episiotomy involving the external anal sphincter (*EAS*). Internal anal sphincter (*IAS*), scarring of perianal tissue (*S*), Vagina (*V*) (reproduced with permission [10])

Episiotomy is a commonly performed surgical procedure in obstetrics. Indications for episiotomy include foetal distress, to expedite the second stage of labour, and assisted vaginal birth. The angle of an episiotomy has a direct bearing on the incidence of obstetric anal sphincter injuries (OASIs). An episiotomy angled to close to the anus can directly injure the anal sphincter complex [1, 2]. An episiotomy angled too far away from the anus does not relieve pressure on the central posterior perineum and is of no benefit in preventing OASIs [3]. Hence, the knowledge and correct practice of episiotomies can minimise avoidable harm by preventing human error (Fig. 1).

In the UK, the National Institute for Health and Care Excellence (NICE) recommends that a mediolateral episiotomy should be performed between 45 and 60° from the midline [4] However, perineal distention causes up to a 30° difference between the episiotomy angle cut and the resulting angle requiring suturing [5]. Therefore, the Royal College of Obstetricians and Gynaecologists (RCOG) specifically recommends a 60° mediolateral episiotomy at crowning [6]. This recommendation is also adopted by the French, Canadian, Saudi and Australian national obstetric guidelines [7]. More recently, a 60° cutting episiotomy angle was adopted by the International Federation of Gynecology and Obstetrics (FIGO) [8]. We could not identify any similar national recommendations in India.

At present, in obstetric practice, clinicians typically perform an episiotomy by visually estimating the required angle or using fixed-angle devices such as the EPISCISSORS-60. However, Andrews et al. [2] showed that no midwife and only 22% of doctors are able to perform a mediolateral episiotomy (between 40 and 60°). Moreover, it has been demonstrated that less than one-third of clinicians are unable to draw a mediolateral episiotomy correctly on paper [9]. This audit was conducted to survey the knowledge and practice of a sample of obstetricians in India regarding the recommended episiotomy angle and their ability to estimate and draw the angle accurately.

## Methods

**Fig. 2:**
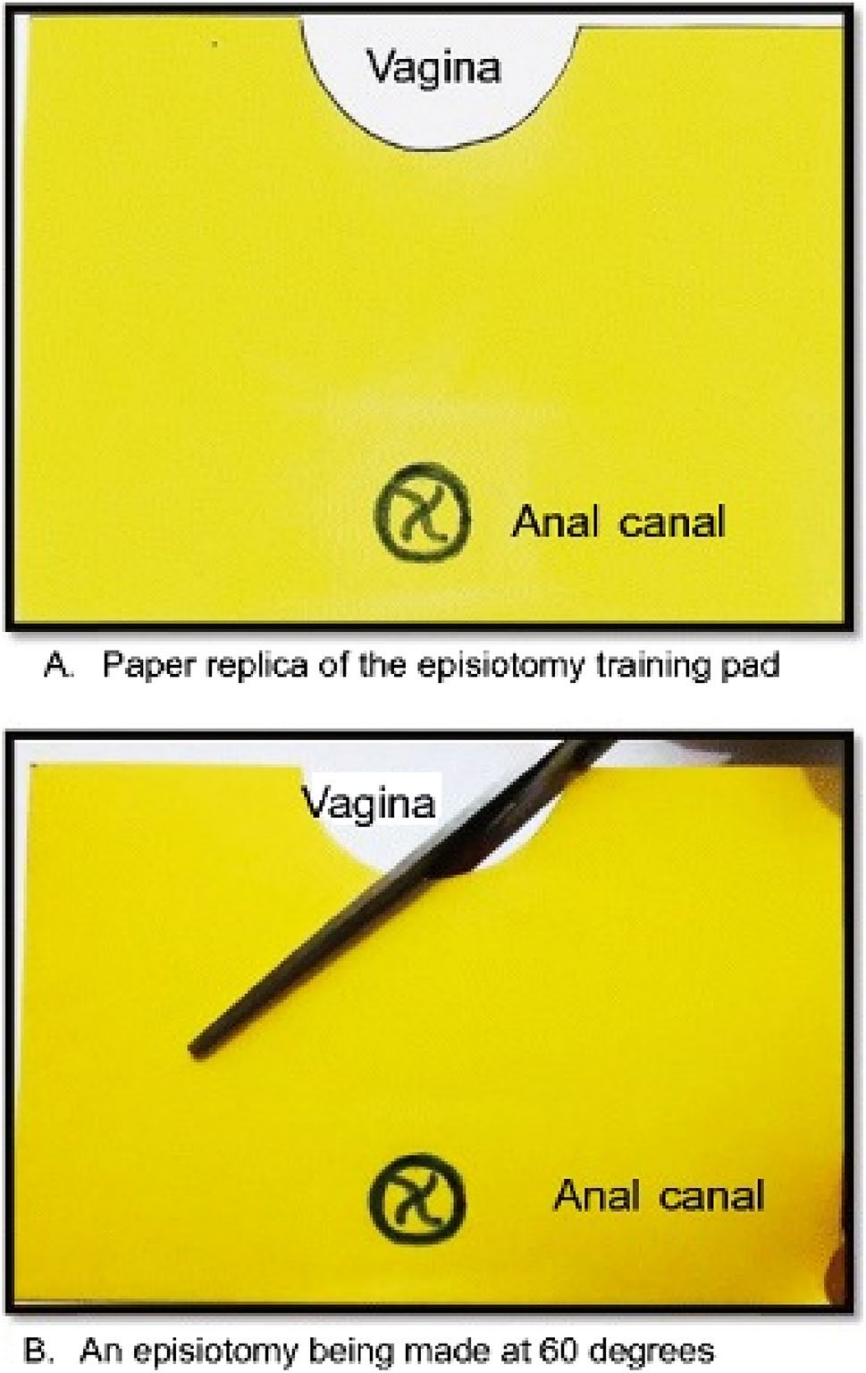
Replica of the perineum on an episiotomy pad showing the angle at which delegates were asked to cut an episiotomy (reproduced with permission [10])

The corresponding author conducted a series of workshops in India as part of the RCOG Sims Black Travelling Professorship award in 2022, with the aim of sharing knowledge in the prevention and repair of OASIs. Doctors from three units in New Delhi, Chennai and Pondicherry were included. The aim of this study was to audit the practice of mediolateral episiotomy in India. Participation in this audit was voluntary. A standard questionnaire was completed by participants prior to the commencement of the workshop. Information gathered included age, gender, height and years of practice since completion of training in obstetrics. Participants were asked to describe the recommended episiotomy angle and the angle was to be drawn on a paper replica of the perineum as described by Naidu et al. [10] (Fig. 2). A right or left cut could be drawn in keeping with the participants’ preference. N.A.O manually measured each episiotomy using a protractor. As this was an audit of clinical practice amongst clinicians, ethical approval and written consent was not required.

Data were analysed using SPSS version 28.0.0.0. Nominal data are expressed as numbers and percentages. The Shapiro–Wilk test was used to check the normality of continuous variables. Continuous data was then reported as median and interquartile range (IQR). Spearman’s rho correlation coefficient was used to assess the difference between continuous variables. To analyse differences in demographic characteristics and episiotomy angle, the Mann–Whitney *U* test or Fisher’s exact test were used where appropriate. Any missing data were excluded from the relevant analysis. The intra-class correlation coefficient (ICC) was calculated to assess the inter-rater reliability between the episiotomy angle stated (the reference standard) and the angle drawn. A ± 2° difference was deemed acceptable. Values of < 0.50 indicated poor, 0.50–0.75 moderate, 0.75–0.90 good and > 0.90 excellent reliability [11]. Standard errors of measurement (SEM) were calculated to measure the range of error of each measurement.

## Results

A total of 140 obstetricians participated in the study. Demographic features are given in Table 1. 90.5% (*n* = 95) described the angle of episiotomy to be between 45 and 60°, and 47.9% (*n* = 67) described the angle of an episiotomy to be specifically 60°. Only 2.2% (*n* = 3) drew the angle of an episiotomy at exactly 60°. However, 8.7% (*n* = 12) drew an angle between 58 and 62° (within the acceptable ± 2° difference) and 15.9% (*n* = 22) drew an angle between 55 and 65° (Table 2). The median episiotomy angle drawn was 52° (IQR = 40–55.3, range 18–77). Increasing years of clinical experience was found to have a weak negative association with the angle of episiotomy drawn (*r*_*s*_ = − 0.199, *p* = 0.02). There was no difference found with regard to age, gender or participant height.

**Table 1:** Demographic details of the included participants

	*N* (N%)/median (IQR)
Age, years (*n* = 133)*	28 (26–32)
Gender (*n* = 140)	
Men	5 (3.6)
Women	135 (96.4)
Height, cm (*n* = 136)*	158 (154–164)
Years of experience (*n* = 132)*	3 (2–6)

*IQR* interquartile range, *N* number

*Missing data

**Table 2:** The number of doctors drawing the episiotomy at different degrees

Angle of episiotomy cut in degrees (°)*	*N* (%)
< 40	33 (23.9)
40–50	52 (37.3)
51–54	17 (12.3)
55–60	12 (8.7)
61–65	10 (7.2)
66–70	11 (8.0)
71–80	3(2.2)
> 80	0 (0.0)

*N* number

*Missing data from two participants (1.4%)

Further analysis was conducted to compare the angle described by the obstetrician and the actual angle drawn by the same person (Table 3). One hundred and three (73.6%) doctors both described and drew an episiotomy angle. Six (5.8%) doctors correctly drew the episiotomy angle they described. There was poor agreement between the angle described and the angel drawn, ICC = 0.18 (− 0.01 to 0.36) with a SEM of ± 12.2°. Of the 67 doctors that described the angle of an episiotomy to be 60°, 0% drew an angle of exactly 60° and 9.0% (*n* = 6) drew a corresponding angle of 60° (± 2°). Participants with incomplete data sets were significantly older in age (28 (IQR 27–33)) in comparison with those with complete data sets (27 (IQR 26–29)). There were no other significant differences with regard to demographic details between the two groups (Table 4).

**Table 3:** Difference in angle described and drawn by doctors

	Median (IQR)/*N* (n%)
Angle described (°)	60 (45–60)
Angle drawn (°)	48 (40–56.5)
Angle comparison (°)*	
Underestimated angle (°)	62 (60.2)
Overestimated angle (°)	35 (34.0)
Correct angle (± 2°)	6 (5.8)

*IQR* interquartile range, *N* number

*Missing data from 37 participants (26.4%)

**Table 4:** Demographic data of participants with missing data and complete data sets

	Missing data: *N*(N%)/median (IQR)	Complete data *N*(N%)/median (IQR)	*p* value
Age, years (*n* = 37)	27 (26–29)	28(27–33)	0.02*
Gender (*n* = 37)			
Men	3 (8.1)	2 (1.9)	0.64**
Women	34 91.9)	101 (98.1)	
Height, cm (*n* = 136)	157 (154–162)	159 (154–165)	0.46*
Years of experience (*n* = 132)	3 (2–5)	3 (2–6)	0.89*

*IQR* interquartile range, *N* number

*Mann–Whitney *U*

** Fishers Exact

Association between the demographic characteristics of the participants and estimation of the episiotomy angle recommended by the RCOG was studied (Table 5). There was no significant association found between any factors including years of experience.

**Table 5:** Association between angle drawn and demographics using the angle recommended by the RCOG

	45–60° *N*(%)/median (IQR)		*p* value
	Yes	No	
Gender (*n* = 138)§			
Male	1 (1.5)	4 (5.5)	0.37*
Female	64 (98.5)	69 (94.5)	
Age, years (*n* = 131)§	28 (27–31)	28 (26–32)	0.74**
Height, cm (*n* = 107)§	157 (153–163)	159 (154–165)	0.11**
Years of experience (*n* = 130)§	3 (2–6)	3 (2–5)	0.85**

*IQR* interquartile range, *N* number

*Fishers exact

**Mann–Whitney *U*

§Missing data

## Discussion

We demonstrated that only 48% of obstetricians had ‘a priori’ knowledge of the recommended angle of the episiotomy (60°) by international peer institutions such as the RCOG and FIGO [6, 8]. In addition, clinicians had poor reliability in estimating episiotomy angle with a potential error of ± 12°.

Strengths of this study include its originality in assessing the reliability of doctors in estimating episiotomy angle. In addition, establishing the range of error of these angle estimations was an advantage. However, limitations of this study should be acknowledged. We acknowledge our sample size was small. Although complete data sets from 74% of the population were obtained, due to resource constraints with regard to the number of units able to be visited, this sample size was anticipated. Due to the nature of survey-based studies, there is a risk of response bias. Furthermore, in some analyses there was up to 26% of data missing due to non-responders, which can introduce selection bias.

Of those who knew the correct episiotomy angle, only 9% were able to draw that angle on a paper replica of the perineum. This confirms the visual inability to estimate angles correctly, as has been shown previously in other studies in the UK, Spain, Norway, Sweden, Denmark, Finland, Iceland and Israel [10, 12–17]. This has more important implications in clinical practice as this may lead to an increase in OASI risk. As the birthing perineum is a convex sphere at crowning, it may be difficult to correctly estimate 60° from the anal midline at this crucial moment. Moreover, there was no accrued benefit from obstetric experience in estimating episiotomy angle. Silf et al. [18] came to the same conclusions in their study in the UK.

Similarly, to our study, the average angle drawn from clinicians in Sweden and Spain was insufficient, with an approximate angle of 50° [13, 17]. In the study by Naidu et al. [10], 106 doctors and midwives in the UK were asked to cut an episiotomy at 60° on a paper replica of the perineum with an episiotomy incision pad. They demonstrated that 15% were able to cut at 58–62° on paper and 36% cut an episiotomy between 55 and 65° range. However, in our study, only 9% drew the angle between 58 and 62° and 16% drew the angle between 55 and 65°. Although Gonzalez-Díaz et al. [17] used a larger range of 50–70°, only 28.1% drew an episiotomy at this angle. Unlike the study by Naidu et al. [10], in our study and that from Gonzalez-Díaz et al. [17], the participants were not told the desired episiotomy angle prior. This further highlights the importance of education surrounding the recommended mediolateral episiotomy angle. We could not identify any Indian national guidelines in this regard. National institutions in India such as the Federation of Obstetrical and Gynaecological Societies of India (FOGSI) would be minded to create good clinical practice guidance in this important issue.

In the study by Ma and Byrd [16], they found the mean angle of episiotomy was 51, 66 and 77° for junior trainees (less than two years’ experience), senior trainees (more than two years’ experience) and consultant obstetricians, respectively. However, there was no analysis performed to establish the difference between these groups [16]. However, we were able to demonstrate that experienced obstetricians were not significantly better in estimating the required angle of an episiotomy than their newer counterparts. Similar findings have also been demonstrated in other studies which found that there was no difference in episiotomy angle with regard to years of clinical experience [13–15, 17]. It has been shown that doctors that have been supervised for at least 10 episiotomies before independent practice are more likely to perform an episiotomy angled further away from the midline [12]. This has implications for creation and implementation of solutions to the problem. To the best of our knowledge, there are no published, validated training programmes that help clinicians improve the visual accuracy of estimation of episiotomy angles. Using fixed-angle devices such as the EPISCISSORS-60 [19], which ensures a cut at 60°, has been shown to reduce episiotomy related OASI by up to 50% in systematic reviews [20–23]. Alternatively, the perineum can be pre-marked at 60° in the second stage with the help of a sterile protractor and a second assistant prior to cutting the episiotomy. This might be difficult to achieve in situations such as foetal distress, where there is an urgency to deliver the baby. The RCOG also clearly stipulates the timing of the episiotomy to be at crowning of the perineum, as earlier episiotomies increase the blood loss. Marking the perineum in the first stage is not accurate as it is prior to the distension of the perineum. Indeed, a 30–45° angle increase has been noted between the first stage and at crowning [24].

## Conclusions

This study has shown that in India, knowledge surrounding the recommended episiotomy angle is lacking. In addition, doctors are not accurate in estimating their desired episiotomy angle. This highlights the need for creating national guidelines for vital intrapartum issues such as the angle and timing of the episiotomy. Structured training programmes to improve visual angle accuracy should be created and validated. Alternatively, fixed-angle devices such as the EPISCISSORS-60 or other proven measurement aids should be used to minimise preventable harm due to human error.
